# Incubation and hatching conditions of laying hen chicks explain a large part of the stress effects from commercial large-scale hatcheries

**DOI:** 10.1016/j.psj.2020.10.015

**Published:** 2020-10-10

**Authors:** Louise Hedlund, Per Jensen

**Affiliations:** IFM Biology, Linköping University, 58183 Linköping, Sweden

**Keywords:** hatchery stress, animal welfare, egg production, White Leghorn, behavior

## Abstract

In commercial egg production, laying hen chicks are exposed to several stressful events during incubation, hatching, and their first hours in life. We have previously shown that hatching and processing are associated with increased corticosterone concentration and further affect behavior and stress sensitivity in a short- as well as long-term perspective. However, it is not known whether these long-term stress effects are caused by the hatchery processing (sex sorting, vaccination, conveying, and loading for transport) or if they are mainly caused by potentially stressful events before processing, during incubation and hatching. In the present study, the aim was to assess the effects of incubation and hatching only, compared to stress effects from the entire hatchery processing. We compared Lohmann LSL chicks incubated, hatched, and processed in a commercial hatchery with chicks incubated and hatched at the same time but not further processed. We studied behavior in a novel arena and during tonic immobility, as well as weight development and corticosterone reaction during a stress challenge. Processed chicks had poorer weight development and were more active in the novel arena test. However, there were no significant differences between the groups in corticosterone reactivity or tonic immobility. When comparing with previous data, both groups had elevated corticosterone concentrations compared to what we had previously reported from chicks hatched under calm and non-stressful conditions. In conclusion, incubation and hatching alone caused long-term stress effects in chickens, but further processing exacerbated these effects to some extent.

## Introduction

In commercial egg production, laying hen chicks are exposed to several stressful events during incubation (such as noise and vibrations) as well as during their first day in life (Hedlund et al., 2019). After hatching, the racks with chicks are taken out of the hatcher and the animals are separated from the shells by hand or by mechanical shaking. The chicks are tilted on a belt system and conveyed through a sex sorting station and a vaccination station to another belt system with accelerating speed and multiple drops. The chicks are then machine counted and dropped into transport boxes which are loaded on to a truck and transported to rearing farms.

The entire process of incubation, hatching, and processing can potentially cause severe stress, and research has shown that stress during early life can have both acute and long-lasting effects on animals. For example, early-life feed restriction affects long-term body weight in *Galliformes* birds such as Japanese quail (Gebhardt-Henrich and Marks, 1995; Hassan et al., 2003), broilers (Plavnik and Hurwitz, 1985; Urdaneta-Rincon and Leeson, 2002), and turkeys (Tůmová et al., 2002). Other types of stressors, such as heat or cold stress, have been shown to result in depressed weight gain (Yahav and Hurwitz, 1996; Altan et al., 2000; Shinder et al., 2002). Some of these effects can be mediated by early priming effects of plasma corticosterone (**CORT**) on the reactivity of the hypothalamic–pituitary–adrenal (**HPA**) axis (Shinder et al., 2002).

Not only factors and events post-hatch, but also many aspects of commercial incubation and hatching may potentially be stressful to the developing chicks. For example, incubation temperature may affect hatch weight (Collin et al., 2005), hatchability (Collin et al., 2005), and plasma CORT concentration in chicken (Yahav et al., 2004), whereas relative humidity affects for example hatchability (Van der Pol et al., 2013), body weight (Bruzual et al., 2000), and embryonic development (Bruzual et al., 2000). In addition, several factors may affect the chick pre-hatch such as light (Deng and Rogers, 2002; Riedstra and Groothuis, 2004; Özkan et al., 2012; Archer, 2018; Yu et al., 2018), noise (Alladi et al., 2005; Chaudhury et al., 2009; Sanyal et al., 2013a; Kesar, 2013; Roy et al., 2014; Donofre et al., 2020), gas exchange (Camm et al., 2001; Rodricks et al., 2004), and mechanical vibrations due to for example transportation (Torma and Kovácsné, 2012; Donofre et al., 2017). Elevated CORT concentrations in the eggs originating from the mother hens can mediate such effects, by for example increasing tonic immobility (**TI**) and decreasing feed intake in the offspring (Eriksen et al., 2003; Janczak et al., 2006).

We have previously shown that chicks have elevated levels of CORT after the hatchery process compared to chicks hatched under calm conditions. The CORT levels were significantly elevated already when the animals were removed from the hatcher, before the processing phase, and then further exacerbated by the processing (Hedlund et al., 2019). This implies that the incubation and hatching environment alone may in fact have contributed significantly to the overall commercial hatching stress. We found that this stress affects the birds in a short- as well as long-term perspective since commercially hatched chicks were more fearful to novelty and had higher CORT reactivity. Later in life, hatchery-processed chicks (**PC**) had more feather damage and injuries on wattle and comb than controls (Hedlund et al., 2019). In the way our previous study was designed, it was not possible to distinguish between stress originating from the post-hatching processing and possible stress inflicted by the incubation and hatching conditions alone. Hence, in the present study, we compared a group of chickens that was obtained from a commercial hatchery after the entire processing with another group that was hatched in the same batch, but did not go through the post-hatch processing procedures.

In the present study, the aim was to assess the effects of commercial incubation alone compared to the stress effects from the entire hatchery processing. Therefore, we compared layer chicks incubated, hatched, and processed in a commercial hatchery according to the regular hatchery procedure, with chicks incubated and hatched at the same time in the same hatchery but not further processed. This allowed us to assess part of the early chick stress that can be attributed to the incubation environment. Since we previously have recorded similar variables as in the present study from chicks hatched under non-commercial, calm conditions (Hedlund et al., 2019), we were also able to discuss our findings in relation to our earlier results in order to assess the specific effects of the commercial hatching conditions.

## Materials and methods

### Ethical Note

All experimental protocols were approved by Linköping Council for Ethical Licensing of Animal Experiments, ethical permit no 14916-2018. Experiments were conducted in accordance with the approved guidelines.

### Animals and Experimental Treatment

All animals were White Leghorn chickens from the Lohmann LSL strain (Lohmann Tierzucht, Germany). In this experiment, 2 groups of animals were used, both containing females as well as males: hatchery hatched and PC and hatchery hatched but non-processed chicks (**N-PC**), both groups described in detail below. All chicks used in the experiment were from the same parental stock placed at the same time in the same egg rack in the same incubator at the commercial hatchery Gimranäs AB, Herrljunga, Sweden. Although males are normally killed and disposed of immediately after hatching, we decided to keep both sexes for this experiment, since possible sex differences in stress responses could add valuable information to help understand the biological consequences of commercial hatchery routines.

#### N-PC

Sixty-five animals (n_f_=38, n_m_=27; subscripts f and m refer to the females and males, respectively) went through the conventional hatchery incubating process which started when fertilized eggs arrived at the hatchery and were placed in a large cabinet incubator. The incubator was calibrated for optimal hatchability with settings described in more detail below. At day 19, the eggs were moved to hatching trays and placed in a hatcher for the last days of incubation. Most eggs hatched at day 21 but according to commercial hatcheries' routines, all eggs and chicks were left in the hatcher until day 22 to maximize hatching rate. N-PC were, when removed from the hatcher, placed in transport cages for a 3.5-hour car transportation to an experimental room at Linköping University. At the university, N-PC were placed in rearing pens and from this point onward were treated in the same way as PC, further described below.

#### PC

Additionally, 79 chicks (n_f_=44, n_m_=35) were incubated and hatched under the same conditions and in the same incubator and hatcher as N-PC. However, at the same time as N-PC were placed in transport boxes, this group was further processed through the conventional hatchery process. The racks with PC were tilted on a conveyer belt and the shells were removed by hand (approximately 3 min). The chicks were then conveyed to a sex sorting station where they were manually sexed by wing inspection (approximately 5 min). As explained above, males were further processed in the same manner as females although this is not according to normal routines. After sex sorting (after which males and females were continued to be treated in the same way), PC were transported via another conveyer belt system to a vaccination station where they were vaccinated against Marek's disease by automatic dispensing machines (approximately 5 min). After vaccination, the animals were moved to a high-speed conveyer belt system with multiple drops in order to spatially separate chicks for efficient machine counting. Chicks were then automatically counted and dropped into transport boxes (approximately 2 min). PC were then transported together with the N-PC in 3.5 h to the experimental room at Linköping University and placed in rearing pens in the same manner as N-PC. From this point onward, PC were treated in the same way as N-PC.

#### Incubator Settings

The incubator and the hatcher at the commercial hatchery were both calibrated for optimal hatchability. The eggs were kept in the incubator for 19 d and were then moved to the hatcher for the last days of incubation. The incubator contained in total 57,024 eggs and the hatcher 19,800 eggs. The eggs were incubated and hatched in complete darkness and in the incubator, but not in the hatcher, the eggs were continuously rotated. In both machines, fans were used to circulate the air in order to maintain the temperature. The noise level of these fans was estimated to 90 dB. At incubation day 0, the temperature in the incubator was set to 37.9 ± 0.1°C and decreased continuously over 18 d to 37.1 ± 0.1°C, while humidity was kept at 30 ± 2%. In the hatcher, the temperature on day 19 was 36.8 ± 0.1°C and it dropped to 36.4 ± 0.1°C at hatch. Humidity was set to 30 ± 2%. During hatching, formaldehyde was evaporated into the hatcher for disinfection purposes.

### Housing

After arrival at the experimental room in Linköping University, the chicks were kept in 4 identical pens, each measuring 90 × 90 cm. N-PC and PC were kept separately but sex mixed in the same groups throughout the whole experiment (pen 1, PC, n_f_=18, n_m_=18; pen 2, N-PC, n_f_=16, n_m_=16; pen 3, PC, n_f_=26, n_m_=17; pen 4, N-PC, n_f_=11, n_m_=22). The floor was littered with wood chips and the chicks were provided with heat lamps, and ad libitum food and water. All chicks had access to perches from 1 wk of age.

### Recordings

#### Behavior in Novel Arena

To assess general fearfulness and explorative behavior, a novel arena test was conducted at the age of 1 d (N-PC, n_f_=10, n_m_=10; PC, n_f_=10, n_m_=10), in the same manner as in our previous study (Hedlund et al., 2019). The arena (57 × 34 × 40 cm) contained an enclosed start box (20 × 20 × 20 cm), sawdust, food, water, and a novel object (a blue glove) to encourage exploration. All birds were, balanced for sex, randomly selected from their home pens and gently carried into the adjacent test room. Two birds, 1 male and 1 female from the same treatment, were tested together and in the statistical analysis the average values of the 2 birds in a pair were treated as 1 replicate. They were placed within the enclosed start box and from the point where the sliding door was opened, behaviors were recorded for 30 min. All the tests were video recorded and behaviors were analyzed afterward. Behaviors recorded were latency to escape the start box and enter the arena, and the total duration of locomotion (2 or more steps of movement) in the arena.

#### TI Test

To assess stress susceptibility, 2 separate tests of TI (Forkman et al., 2007) were performed, 1 baseline and 1 immediately preceded by a brief event of acute stress. The baseline TI test (**TI**_**b**_) was conducted at the age of 1 d (N-PC, n_f_=10, n_m_=10; PC, n_f_=10, n_m_=10). Individuals used in novel arena were not used in TI. All chickens, balanced for sex, were selected randomly, and carried one at a time into an adjacent test room. The bird was placed on its back in a cradle and a light pressure was applied to the body for 10 s. Chickens that righted within 5 s were regarded not to have entered TI and the process was repeated up to a maximum of 3 times. Chickens that did not enter TI were excluded from the analysis. All tests were performed by the same person. Time of first vocalization, time of first head movement, and time of rightening were recorded as well as vocalization frequency.

Three days later, at 4 d of age, a second TI test was performed. This test was performed on the same birds and according to the setup for TI_b_; however, in this second TI test, the chickens were prestressed, in order to assess any differences in stress susceptibility between the groups. The birds were carried from the home pen and were socially isolated in a box (57 × 34 × 40 cm) with solid walls, a sawdust-covered floor, and a wire net on top. Social isolation is a well-known stressor to young chicks (Hymel and Sufka, 2012). After 2 min, each bird was gently picked up and placed in the cradle in the same manner as in TI_b_. The same behaviors as in TI_b_ were recorded.

#### HPA-Axis Reactivity

To assess reactivity of the HPA axis, a restraint test was conducted at 6 d of age (N-PC, n = 11; PC, n = 11). Individuals already tested in novel arena or TI were not used in the restraint test. Chicks were selected randomly from their home pen and a blood sample was taken from the wing vein within 3 min of capture in order to establish a baseline concentration of CORT (CORT_b_). The chickens were then restrained in a net bag for 3 min before a second blood sample was taken for measuring CORT increase after the stressor (**CORT**_**s**_).

All blood samples were collected using B Braun Sterican Syringe Needles (B Braun, Melsungen, Germany) and Microvette heparin-coated tubes (200 μL, Sarstedt Inc, Newton, NC). The blood samples were stored in a refrigerator and centrifuged in the laboratory later on the same day. Plasma was separated, frozen and stored at −20°C until the time of analysis. For the analysis, a corresponding ELISA test from Enzo Life Sciences (Farmingdale, NY) was performed. Normal protocol was used and the samples were measured in duplicate and analyzed according to the product manual: http://static.enzolifescience.com/fileadmin/files/manual/ADI-900-097_insert.pdf.

#### Weight

A random sample of chicks (Table 1) was weighed at 1, 7, 14, 21, and 28 d of age. They were taken from their home pens and weighed individually on a balance with a precision of 0.01 g.

**Table 1:** Mean weight in PC and chickens only incubated (N-PC), in grams, in males and females, from 1 to 28 d of age.

Age (d)	PC		N-PC		Sex			Treatment		
	m	f	m	f	χ^2^	df	*P*-value	χ^2^	df	*P*-value
1	42.2 ± 0.7 (n = 20)	42.4 ± 0.7 (n = 20)	44.8 ± 0.8 (n = 19)	43.4 ± 0.7 (n = 20)	0.611	1	0.434	5.648	1	0.017
7	72.1 ± 2.0 (n = 9)	68.9 ± 2.1 (n = 8)	72.3 ± 1.8 (n = 11)	66.9 ± 2.7 (n = 5)	3.598	1	0.058	0.086	1	0.770
14	124.4 ± 1.8 (n = 35)	119.2 ± 1.9 (n = 34)	128.9 ± 1.8 (n = 37)	123.4 ± 2.2 (n = 25)	7.674	1	0.006	5.232	1	0.022
21	203.8 ± 3.1 (n = 32)	184.0 ± 2.7 (n = 42)	211.2 ± 3.0 (n = 36)	199.0 ± 3.6 (n = 25)	27.547	1	<0.001	12.504	1	<0.001
28	303.6 ± 3.9 (n = 35)	268.6 ± 3.7 (n = 38)	306.2 ± 3.8 (n = 36)	278.3 ± 4.6 (n = 25)	62.988	1	<0.001	2.143	1	0.143

Abbreviations: f, females; m, males; N-PC, non-processed chickens; PC, hatchery-processed chickens.

### Statistical Analysis

The weight data were analyzed with a generalized linear model with sex and treatment as factors, using the normal distribution function and the link function “identity.” There was no effect of interaction between the factors, and therefore this was excluded from the later models. For the behaviors in the novel arena test, we used a similar generalized linear model and regarded the data of the 2 birds tested together in 1 arena as 1 replicate. For the TI as well as the HPA-axis reactivity results, generalized linear mixed models with repeated measures were used to analyze differences between PC and N-PC. In both these cases, there was no effect of sex and therefore it was removed from both analyses. All the statistical analyses were carried out in SPSS (SPSS Inc., Chicago, IL).

## Results

### Novel Arena

**Figure 1:**
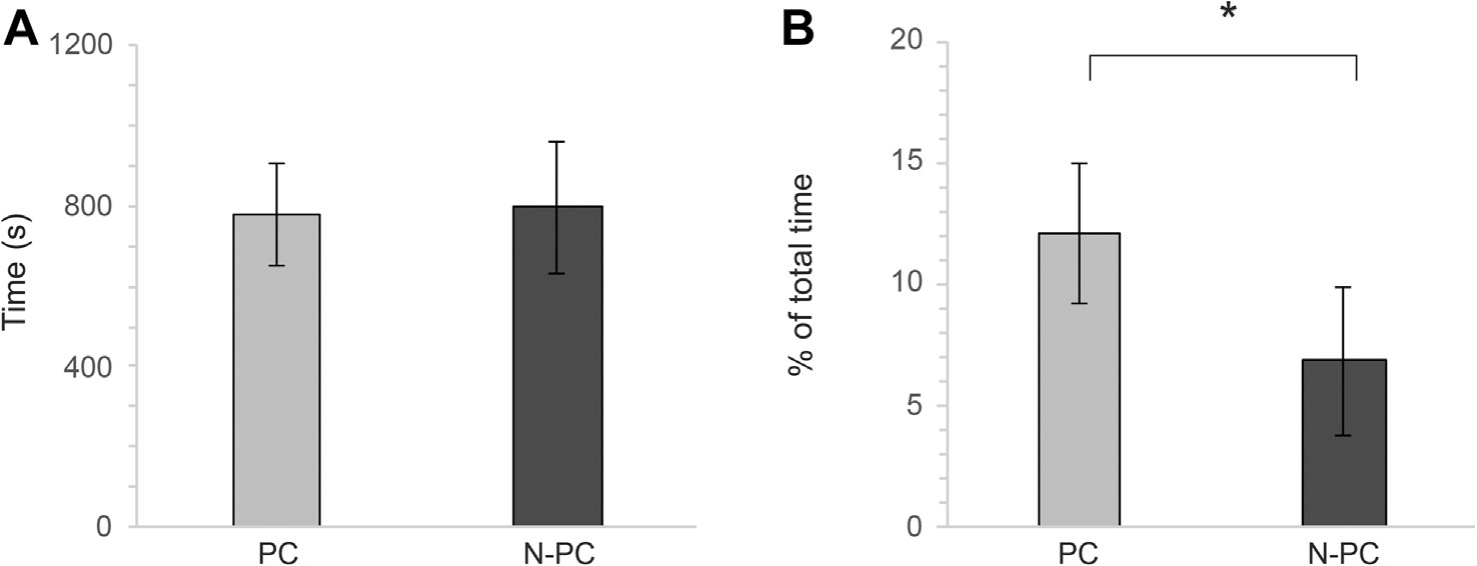
Behaviors of PC and N-PC in novel arena. (A) Latency to emerge from the start box and enter the arena, in seconds. (B) Locomotion behavior in novel arena, % of total time spent outside the start box in the arena. ∗*P* < 0.05. Abbreviations: N-PC, non-processed chickens; PC, hatchery-processed chickens.

Novel arena testing showed that there were no differences between treatments in latency to escape the start box and enter the arena (χ^2^ = 0.075, df = 1, *P* = 0.785, Figure 1A). However, there was a difference in activity in the arena where PC were more active than N-PC (χ^2^ = 3.960, df = 1, *P* = 0.047, Figure 1B).

### TI

**Figure 2:**
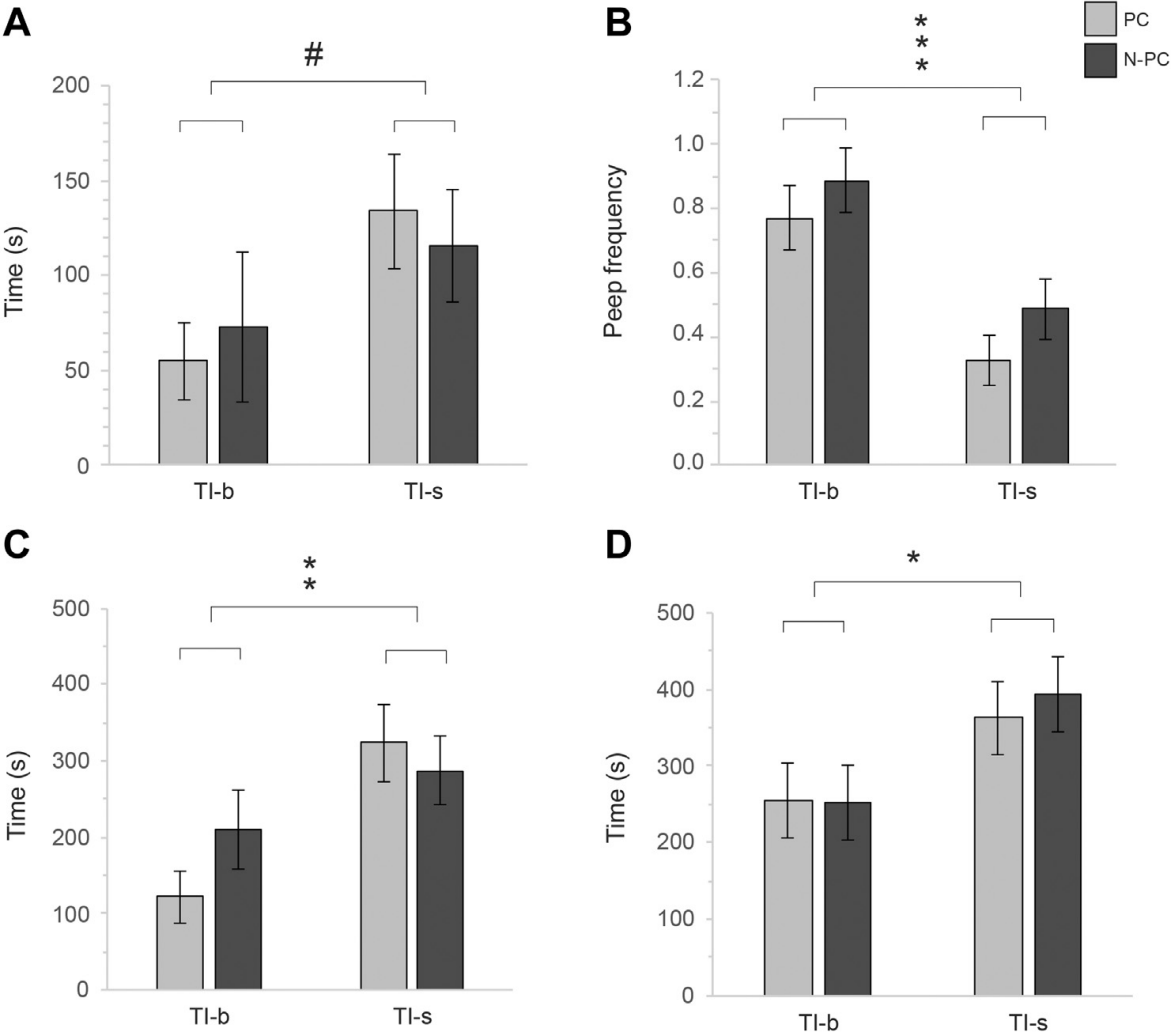
Behaviors of PC and N-PC during tonic immobility: (A) latency to first peep; (B) peep frequency; (C) latency to first head movement; (D) latency to rightening. ^#^*P* = 0.057, ∗*P* < 0.05, ∗∗*P* < 0.005, ∗∗∗*P* < 0.001. Abbreviations: N-PC, non-processed chickens; PC, hatchery-processed chickens; TI_b_, baseline tonic immobility test; Ti_s_, tonic immobility test after acute stress.

Isolation stress had a significant (or almost significant) effect on all variables in the TI test: latency to first peep increased following stress (F_1, 75.795_ = 3.735, *P* = 0.057, Figure 2A), while the frequency of peeps decreased (F_1, 73.889_ = 19.349, *P* < 0.001, Figure 2B). Latency to the first head movement increased (F_1, 75.578_ = 8.666, *P* = 0.004, Figure 2C), as did the rightening time (F_1, 75.968_ = 6.137, *P* = 0.015, Figure 2D). However, there were no effects of the hatchery treatment (PC vs. N-PC) on any of the variables and no significant effects of the interaction between hatchery treatment and isolation stress.

### HPA-Axis Reactivity

**Figure 3:**
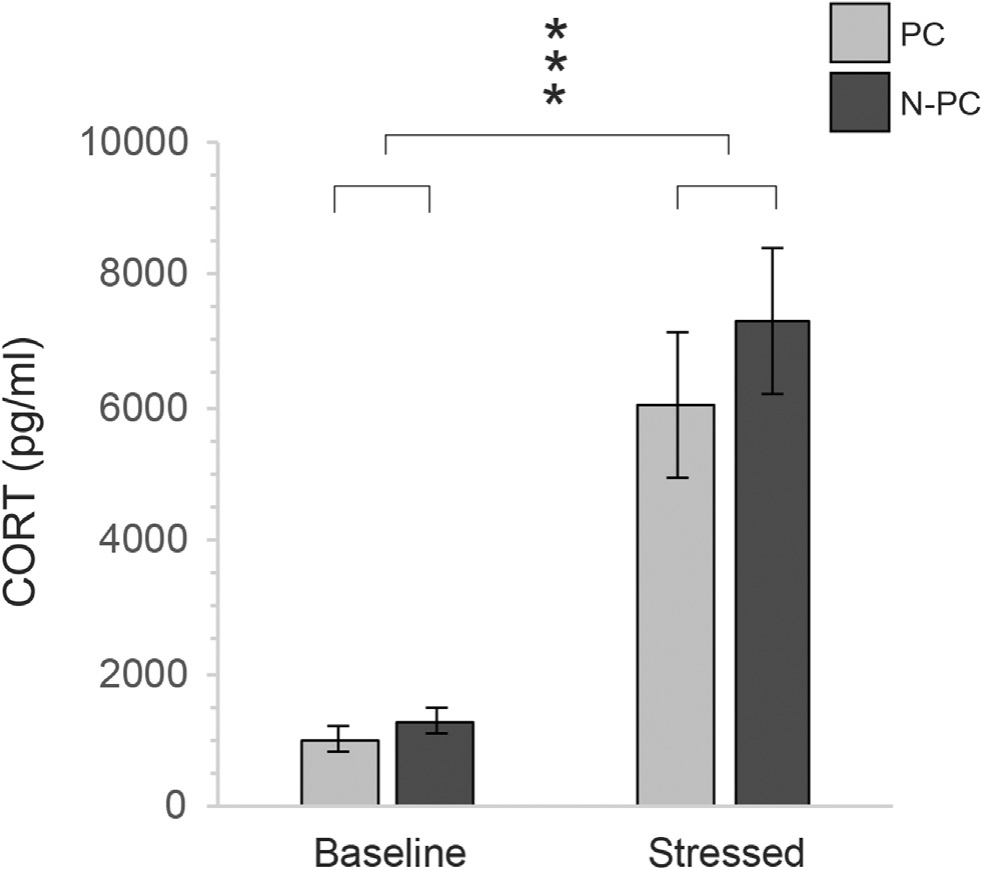
CORT concentration in PC and N-PC before and after 3 min of restraint, ∗∗∗*P* < 0.001. Abbreviations: CORT, corticosterone; N-PC, non-processed chickens; PC, hatchery-processed chickens.

For the HPA-axis reactivity test, there was a significant difference between CORT_b_ and CORT_s_ showing that the restraint caused an increase in the hormone level (F_1, 21.126_ = 48.567, *P* < 0.001, Figure 3). However, there was no significant effect of hatchery treatment on the CORT increase after stress (treatment, F_1, 21.126_ = 0.909, *P* = 0.351; treatment∗restraint, F_1, 21.126_ = 0.392, *P* = 0.538; Figure 3).

### Weight

N-PC birds were heavier than PC birds at 1, 14, and 21 d of age (Table 1). However, there was no difference between the treatment groups on days 7 and 28. From 7 d of age, males were significantly heavier than females, and as mentioned previously, the interaction term was dropped from the model since it had no significant effect.

## Discussion

Our results showed that there are relatively few differences in behavior and stress responses between chicks that were subjected to the entire commercial hatchery procedure compared to chicks that were only incubated and hatched, but not further processed, in the same hatchery. This indicates that a major part of the previously demonstrated long-term stress effects of commercial hatching (Hedlund et al., 2019) may be caused by incubation and the hatch environment. However, the rest of the processing (sex-sorting, vaccination, conveying, etc.) appears to exacerbate the long-term consequences to some extent, as shown by the fact that processed birds have a reduced weight up to 3 wk of age. The present results are in line with our previous findings, where chicks showed elevated concentrations of CORT immediately after hatching compared to chicks hatched under calm control conditions (Hedlund et al., 2019).

When challenged with a novel environment, PC were significantly more active than N-PC. This implies that N-PC were more fearful than PC since reduced activity is considered to be a validated fear response in chicks (Jones and Waddington, 1992; Forkman et al., 2007). There was no difference between the groups in latency to enter the arena. The reduced fear response in N-PC is difficult to explain; however, possibly, the handling during the processing part may have primed them to show less fear. Previously, we found that chicks that were both hatched and handled under calm, non-stressful conditions were more active in the same test than chicks that had been hatched and processed in a commercial hatchery (Hedlund et al., 2019). However, in the present experiment, the data for both PC and N-PC were closer to those of hatchery PC than to the control group in the previous test. Since the experimental setup was exactly the same in both experiments, this suggests that the increased fearfulness observed in hatchery PC in our previous study was largely caused by the incubation conditions.

Tonic immobility is a well-validated fear test in chickens that has been found to correlate with other fear tests (Forkman et al., 2007). In this study, there were significant effects of a brief isolation stress on all the variables measured, in accordance with previous research (Jones, 1992; Marin et al., 2001), although we acknowledge this might also be due to the second round of testing or age. However, we did not find any differences between PC and N-PC, which suggests that the hatchery processing did not increase the fear responses measured in TI compared to incubation only, and also did not affect the stress susceptibility.

Hypothalamic–pituitary–adrenal axis reactivity was tested with a restraint test and showed no difference between the treatments in this study. However, the CORT concentrations of the birds after restraint were about 3 times as high as for the control chicks in our previous study (Hedlund et al., 2019) and the levels were very similar to the hatchery-processed birds in that experiment. Again, the results imply that the incubation and hatch conditions are responsible mainly for the long-term consequences in stress susceptibility, since the processing after hatching did not further increase the CORT reactivity.

Although the processing part of the commercial hatchery routine did not lead to major changes in the behavior or the HPA-axis reactivity compared to chicks that had only experienced the incubation and hatching part, our results did show that processing does have some exacerbating effects. We found a difference in weight wherein N-PC weighed more than PC, an effect that lasted until 3 wk of age. We have previously demonstrated that CORT concentration, although being elevated already after incubation, increases further during the hatchery processing (Hedlund et al., 2019). This might be one of the factors affecting the weight differences. Previous research has shown that early exposure of CORT may have negative influences on body weight and growth (Eid et al., 2003; Eriksen et al., 2003; Janczak et al., 2006; Lin et al., 2006) and can change diet preference in chickens (Malheiros et al., 2003).

When comparing the results from this study with our previous work (Hedlund et al., 2019), we find support for that these are related to hatchery stress. However, we acknowledge that the sample size in this study was roughly half the size of the previous one and the results should be compared with that in mind. Furthermore, the birds were all hatched in the same hatcher, and it cannot be excluded that this was not representative for all the hatchers in the hatchery. However, hatching conditions are carefully monitored by the operators, and there were no indications of any deviations from normal in this batch. A further caveat is that we had few pen replicates, which also calls for some care in the conclusions drawn from our study. However, the fact that both hormone concentrations and behavior of the chicks were very similar to what we previously observed from hatchery chicks, makes it highly probable that the present findings represent results of stress encountered during incubation and hatching.

In summary, we found that the previously reported long-term effects of commercial hatchery processing on behavior and HPA-axis reactivity (Hedlund et al., 2019) can be largely attributed to the stress experienced during incubation and hatching. Possibly, the stress during incubation and hatching may be so intense that further handling and processing—although stressful in its own right—does not add substantially to the long-term effects.

Many factors can affect chicks pre-hatch, for example noise (Alladi et al., 2005; Chaudhury et al., 2009; Sanyal et al., 2013a; Kesar, 2014; Roy et al., 2014; Donofre et al., 2020), light (Riedstra and Groothuis, 2004; Özkan et al., 2012; Archer, 2018; Yu et al., 2018), high amounts of CORT (Eriksen et al., 2003; Janczak et al., 2006), reduced gas exchange (Camm et al., 2001; Rodricks et al., 2004), and mechanical vibrations due to for example transportation (Torma and Kovácsné, 2012; Donofre et al., 2017). In a commercial hatchery incubator, the temperature and the humidity are highly controlled, and chicks are incubated in complete darkness. However, one important factor that might increase stress and affect the welfare of the chicks is the noise caused by fans in the incubator which can reach levels of about 90 dB (Donofre et al., 2020). It is well known that chicken embryos can detect and respond to external sound from day 16 of embryonic development (Jones et al., 2006; Roy et al., 2014), although it is argued that hearing starts to develop as early as day 12 of incubation, since the formation of the hair cell afferent synapses takes place around this time point (Jones et al., 2006). In parallel with the hearing, the HPA axis starts developing early in the chicken embryo. Adrenocorticotropic hormone has been detected already at day 7 of embryonic development and neurons in the hypothalamus that secrete corticotropin releasing hormone, which control adrenocorticotropic hormone secretion from the pituitary gland, have been detected at around 14 d of incubation (Jozsa et al., 1979). There seem to be 2 sensitive periods where plasma cortisol concentrations in the chick embryo increase distinctively: day 14 to 16 of incubation and immediately before hatch (Scott et al., 1981; Tanabe et al., 1986).

Research has shown that loud noise during this later half of incubation can increase plasma noradrenaline levels (Sanyal et al., 2013a), impair spatial behavior (Sanyal et al., 2013a), and decrease body weight (Kesar, 2014) as well as brain weight (Sanyal et al., 2013b; Kesar, 2013, 2014) and size (Kesar, 2014). On the other hand, patterned music and species-specific patterned sounds can positively modulate spatial orientation, learning, and memory (Sanyal et al., 2013a), increase total volume of the brain (Sanyal et al., 2013b), and increase synaptic density in parts of the hippocampus which suggest strengthening hippocampal function (Chaudhury et al., 2009).

In addition, in many commercial hatcheries (including the one we studied), formalin is evaporated; so chicks are exposed to this gas during the last 3 d of incubation and about 1 d after hatching. Although not properly investigated, this highly noxious gas may have several effects on further development and welfare of the chicks (Sander et al., 1995; Fauziah et al., 1996; Zulkifli et al., 1999; Cadirci, 2009), but clearly, more research is needed on this aspect.

## Conclusions

In conclusion, we found that chicks hatched and processed in a commercial hatchery (PC) differed from chicks that were only incubated and not further processed (N-PC) in some important aspects. PC weighed less and were more active in a novel arena test than N-PC, possibly as a result of the additional stress inflicted by post-hatch processing. However, there were no differences between PC and N-PC in latency to enter a novel arena, behavior in TI, or CORT reaction to stress. In these respects, both groups deviated substantially from earlier studied birds that were incubated and hatched under calm control conditions. The fact that both the hatchery-processed and the incubated-only chicks showed similar levels of stress responses suggests that a large part of the stress experienced in commercial hatcheries is caused by the incubation and hatch conditions rather than the processing itself.
